# Emerging Biomarkers and Therapeutic Strategies for Refractory Bullous Pemphigoid

**DOI:** 10.3389/fimmu.2021.718073

**Published:** 2021-08-24

**Authors:** Tong Zhou, Bin Peng, Songmei Geng

**Affiliations:** Department of Dermatology, The Second Affiliated Hospital, School of Medicine, Xi’an Jiaotong University, Xi’an, China

**Keywords:** bullous pemphigoid, biomarkers, severity, refractory, relapse, prognosis, biologics

## Abstract

Bullous pemphigoid (BP) is the most common autoimmune subepidermal blistering disorder in the elderly. Systemic and topical use of glucocorticoids and immunosuppressants has been shown to be effective in most patients. However, refractory BP patients are challenged to clinicians with severe clinical symptoms, resistance to treatment, and high relapse rate. How to predict and assess the refractory and severity of bullous pemphigoid is the key issue in clinical practice, and the urgent need for precision medicine in refractory patients is driving the search for biomarkers and biologics. Recently, some biomarkers, such as the level of specific autoantibodies and released cytokines, have been proposed as the potential parameters to reflect the disease severity and predict the treatment response and relapse of refractory BP. Moreover, new biologics targeting pathogenic antibodies, complement, Th2 axis, eosinophils, and Th17 axis have shown potent efficacy on refractory BP. Here, we review the literature and give an overview of emerging biomarkers and therapeutic strategies for refractory bullous pemphigoid to improve the prognosis of the patient.

## Introduction

Bullous pemphigoid (BP) is an uncommon autoimmune subepidermal blistering disease, but accounts for about 70% of subepidermal bullous diseases, mainly affecting the elderly (1). It is estimated that the annual incidence of BP among different populations in the world is about 12–66 cases per million people (2). The typical clinical features of BP consist tension blisters on erythema or normal skin with intense itching. Histopathology shows subepidermal blisters and inflammatory cell infiltration dominated by eosinophils. Immunologically, IgG and/or C3 are deposited linearly along the basement membrane zone, characterized by the production of autoantibodies against the components of the hemidesmosomes BP180 and BP230 at the dermis-epidermis junction (3).

**Figure 1:**
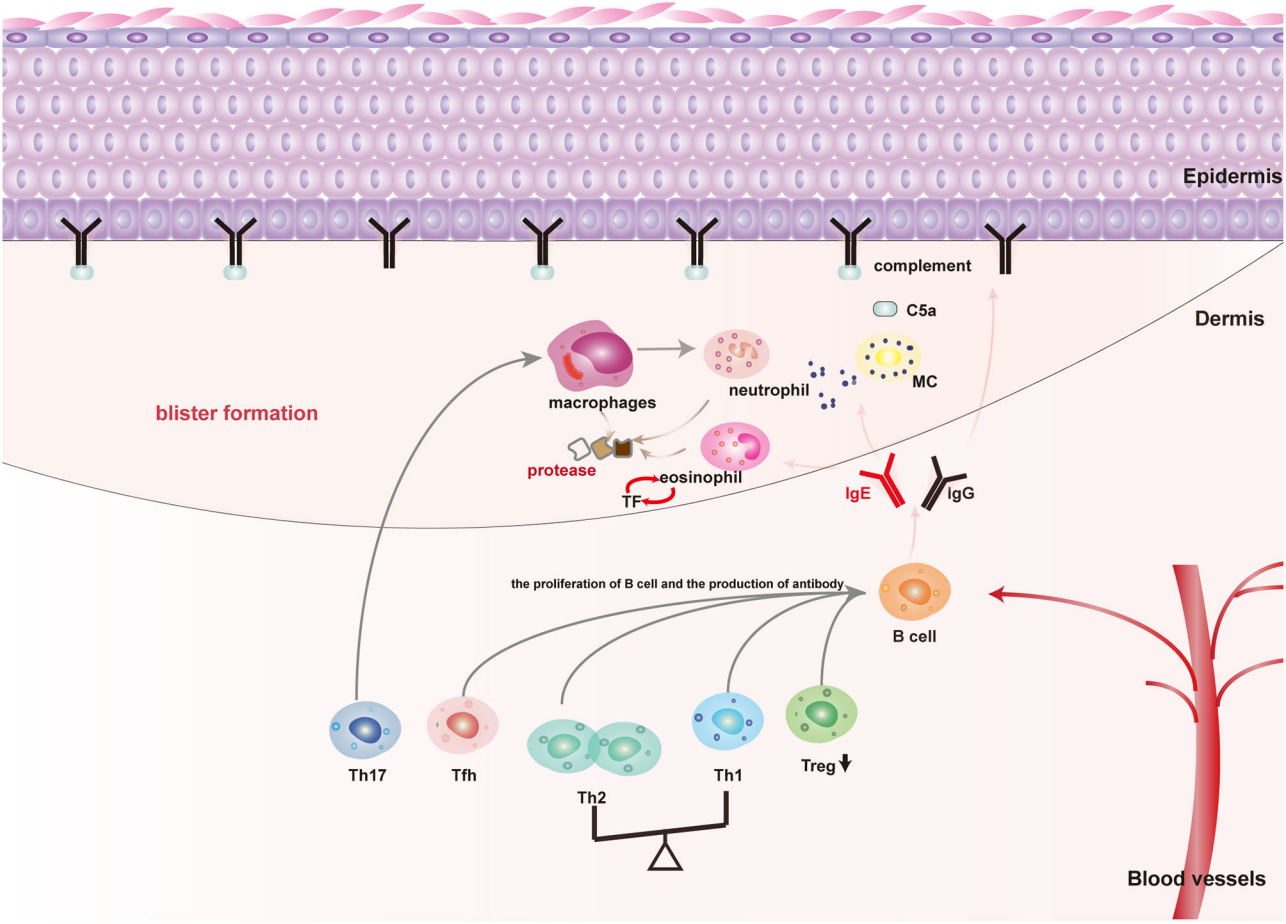
The main pathogenic mechanism of blister formation in patients with BP: Autoimmune abnormalities such as dysfunction of Th cells and Treg cells lead to the production of anti-BP180 autoantibodies, among which BP180IgG and IgE are the main pathogenic antibodies for blister formation. (1) Mechanical damage: Both BP180IgG and IgE could bind to the NC16A domain of BP180, resulting in BP180 internalization, thus decreasing the adhesion; (2) Inflammatory injury: BP180IgG and IgE could also activate keratinocytes to release IL-8 and other cytokines by binding to BP180. Moreover, BP180IgG and IgE also activate mast cells in a complement-dependent or independent way to release cytokines and mMCP-4. These above cytokines and mMCP-4 recruit and activate inflammatory cells dominated by eosinophils and neutrophils to magnify the inflammatory response of skin lesions and cause tissue damage; (3) Specific pathways of mast cell activation: BP180IgG and IgE directly activate mast cells by binding to FcγR or FcϵR of mast cells. BP180IgG binding BP180 also activate the complement cascade pathway to produce C5a, which binds to the C5aR of mast cells and indirectly causes mast cell activation.

Genetic predisposing factors, such as HLA-DQβ1^∗^0301 is associated to the occurrence of BP, UV exposure, thermal or electrical burns, trauma, drugs, virus infection, and other physical, chemical, and biological factors that are involved in the pathogenesis of susceptible individuals by mediating the loss of immune tolerance to the autoantigen (4). In terms of the pathological mechanism of BP, autoantibodies, immune cells, and inflammatory factors are all involved in the pathogenesis of BP. The interaction of autoantibodies with BP180 brings about the formation of blisters by the activating complement-dependent or independent signals to amplify the inflammatory pathway (**Figure 1**) (5). Non-specific immunosuppressive therapy is effective in most patients. Unfortunately, refractory BP patients are resistant to glucocorticoids or immunosuppressants treatment or have a high relapse rate of 27.87%–53%, and most of the relapse occurs in the early stage of remission (within 6 months) (6). Besides, severe adverse reactions due to a long-term use of glucocorticoids or immunosuppressants, including infections, gastrointestinal intolerance, myelosuppression, hepatotoxicity, and even increased risk of cancer could not be ignored (7–9). Hereby, biomarkers for predicting the refractory BP and indicating optimal therapeutic strategies to control their symptoms are urgently required.

With a deep understanding of the pathogenesis of BP, several studies have reported that some parameters involved in the pathogenesis can be used as biomarkers for refractory BP. The purpose of this paper is to analyze the relationship between biomarkers and the disease activity, relapse, and response to treatment. Moreover, the clinical application of new biologics for refractory BP would be reviewed.

## Emerging Biomarkers in BP

### Anti-BP lgG and lgE

The reaction of specific autoantibodies produced by autoimmune abnormalities with BP180 is regarded as the central event in the pathogenesis of BP. Both anti-BP180 IgG and IgE could reduce the adhesion of keratinocytes by increasing the internalization of BP180 as well as mediating the recruitment and activation of immune cells through complement-dependent or complement-independent pathways to release proteases and cytokines, thus participating in the formation of blisters (5).

#### BP180 IgG

BP180 ELISA values and direct immune fluorescence (DIF) are necessary to diagnose BP. The value of anti-BP180NC16A IgG ELISA is positive correlate with the disease severity in cross-sectional studies (10–15). The longitudinal follow-up of large samples during traditional treatment suggests that patients with a high level of BP180NC16A IgG indicate refractory BP who have an insufficient response to glucocorticoid and requiring a longer treatment time or a higher dose of glucocorticoid (12, 15–18).

Moreover, BP180IgG levels are of great clinical importance in predicting the risk of relapse. High titer of anti-BP180NC16AIgG (>27 IU/mL) is the only factor independently predicting the relapse of BP within one year after discontinuation of treatment, and the positive predictive value and the negative predictive value are 90.9% and 51.2%, respectively (19). However, in another study, positive DIF was of a better value than BP180 IgG ELISA values for predicting the relapse risk, with 59.0% sensitivity, 78.0% specificity, while 50.0% positive predictive values and 83.3% negative predictive value, respectively (20). When the ELISA-BP180 value was high or DIF shows positive, treatment to BP should not be ceased. Relapse is concerned with a slight deceleration of BP180 IgG levels between day 0 and day 60. A high level of BP180 at day 150 (23 U/mL) (21) and baseline (53.09 U/mL) could also predict relapse, the sensitivity was 84.2% and 81.3%, respectively. Meanwhile, the negative reaction to BP180 IgG and BP230 IgG ELISA may be a clue of non-relapse (22).

It should be noted that about 8% of BP cases have anti-BP230 but not anti-BP180 (BP230-type BP). Most studies showed that anti-BP230IgG levels was not related to disease severity in the presence of BP180 IgG (3). A large proportion of BP230-type cases showed a mild clinical phenotype, which may be related to the weak deposition of IgG1, IgG3, and complement in lesions, indicating the importance of IgG antibody subclass analysis (23). Consistently, as a blocking antibody, it is found that the IgG4 level increased with the improvement of the disease condition, so the change of the main antibody subclass from IgG1 to IgG4 may mean the improvement of the disease (24).

#### BP180 IgE

Recently, increasing evidence has shown that IgE is involved in the occurrence of BP, but contradictions are still in the relationship between IgE and disease severity, which may be due to the lack of a unified method for the quantitative detection of BP180IgE (3). Elevated IgE levels have been reported in 22%–100% of patients with BP (25). A few studies reported that the circulating level of BP180NC16A IgE in patients was not related to the severity of the disease (26–28). For example, Ma et al. believed that high titers of anti-BP180 IgG contributes mostly to the disease activity than the anti-BP180 IgE level in patients (28). However, since previous studies have verified the pathogenic effect of BP180IgE in cell culture and animal models, Omalizumab targeting IgE has been successfully used in patients with refractory BP (3). It agrees with the conclusion that high levels of circulating BP180NC16A IgE and total IgE in serum are correlated with higher BPDAI scores of BP patients (11, 13, 25, 29–32). It is an urgent need to explore and unify BP180 IgE detection methods with acceptable sensitivity and specificity to confirm this conclusion. On the other hand, as a low-affinity Fc receptor of IgE, the levels of CD23 in tissue or and sCD23 in serum are consistent with the severity of the disease (33–35), which also imply that IgE is another key participant in the pathogenesis of BP. In addition, based on the existence of high levels of anti-BP180IgG antibodies, the appearance of anti-BP180IgE supports that those patients need more active therapies for remission (31). The patients with a high level of anti-BP180 IgG need to jointly evaluate the level of anti-BP180 IgE in a subsequent treatment to more effectively identify refractory BP patients. Not only that, when the total serum IgE level remains high and the skin lesions could not be effectively controlled, Omalizumab can be considered (13, 36–38).

### Chemokines

All infiltrating cells contribute to the pathogenesis of BP. Especially, the chemotaxis and activation of major effector cells such as eosinophils, neutrophils, and monocytes/macrophages in the lesions, directly leading to the blister formation through the release of proteases and cytokines (39). Chemotactic factors that attract inflammatory cells infiltrating should be attended to.

CXCL8 (IL-8), a neutrophil-attracting chemokine, in serum and blister fluid (BF) of patients could change with the fluctuation of the disease activity (40–42). CXCL9(MIG), CXCL10 (IP10), and Th2 chemokine, such as CCL2 (MCP-1), CCL17 (TARC), CCL18 (PARC), and CCL22 (MDC) cannot only attract T lymphocytes and monocytes to the lesion but also relate to the disease severity (43–48). In addition, elevated serum CXCL9 levels only occur in patients with a serious condition, and should be particularly vigilant against the disease deterioration (44, 47). CXCL10 induces monocytes and neutrophils to express MMP-9, which is involved in the relapse of BP. The serum CXCL10 levels can be upregulated by IL-17 and continue to rise or maintain high levels at 60 days or within the first year after treatment in relapse patients, which maintain and amplify the inflammatory response (49). What is more, CCL17 levels sharply reflect the disease activity with a higher sensitivity than BP180NC16A IgG, and could be viewed as a promising biomarker for predicting the early relapse of the disease (43).

Cytokines such as IL-5, CCL5 (RANTES), CCL11 (Eotaxin), and CCL26 (Eotaxin3) are involved in the proliferation and recruitment of eosinophils from the peripheral circulation to the lesions, and their levels always keep in line with the number of blisters (40, 44, 50–52). Particularly, IL-5 is the promising biomarker for disease severity, as IL-5 levels in serum and BF could reflect the disease severity in several different population studies. The count of peripheral blood eosinophils fluctuates in line with the disease severity in BP (30, 32, 53–58).

### Cytokines

Inflammatory mediators, for instance, IL-1β, IL-6, and TNF-α, reflecting the disease severity, because serum levels of TNF-α, IL-6 and blister contents of IL-1β and TNF-α are significantly higher in severe patients than in mild patients. But they are not specific enough for BP, because they rise in many other conditions (40–42, 59). In contrast, patients with severe clinical manifestations and treatment resistance are often accompanied by low levels of IL-10 secreted by Treg, and the increasing levels of IL-10 is related to clinical improvement in BP (60–62). These observations support the idea that a persistent immune activation exists in diseases that are relatively or absolutely deficient in IL-10, and the recovery of relatively defective Treg and IL-10 is helpful to alleviate the disease.

Moreover, some members of the TNF family, such as sCD40L, a proliferation-inducing ligand (APRIL), and B lymphocyte activating factor (BAFF), contribute to B cell proliferation and autoantibodies production, they are more sensitive than autoantibodies to reflect the treatment response and early relapse. They often occur before the appearance of skin lesions and the increase of BP180NC16A antibody levels, and decline after treatment, so that detection of their serum levels could distinguish the tendency of relapse in time (63–65). Likewise, IL-18, as well as IL-15 derived from macrophages and IL-21 secreted by Tfh cell increase in BF or serum of BP, which promote the proliferation of B cells and the production of antibodies, also correlate with the disease activity (42, 66–68).

In BP patients with a continuous remission and relapse in the first year of treatment, serum autoantibodies titers, as well as concentrations of cytokines belonging to the Th17 axis are different. IL-17 and IL-23 participate in a variety of pathophysiological mechanisms of relapse. To begin with, both IL-17 and IL-23 independently induce DNA extracellular traps (ETs) formation in patients with relapse; subsequently, they also stimulate monocytes and neutrophils to produce MMP-9, which contributes to the separation of the dermis and epidermis; eventually, they upregulate the expression of glucocorticoid receptor-β related to the resistance to glucocorticoid therapy (69, 70). During the one-year follow-up, Plée et al. found that the patients with a significantly decreased IL17 level within 0–60 days showed a continuous remission, while the patients whose IL-17 level remained high within 60 days after treatment had a higher risk of relapse. Similarly, patients with significantly elevated IL-23 and MMP-9 levels within 0–60 days were also prone to relapse (69).

However, we have doubts about the predictive value of serum inflammatory mediator levels not only because a variety of factors influence the inflammatory mediator levels in serum and make them unrepresentative of the levels in tissues, but because the dynamic monitoring of serum inflammatory mediator levels is costly.

### Eosinophil and Eosinophil Cationic Protein

Eosinophils, as the main infiltration cell in the lesions of BP, play the central effect on blister formation and pruritus by secreting protease and cytokines like Eosinophil cationic protein (ECP) and IL-31 (3), it also provides several biomarkers to reflect disease severity and relapse.

ECP in BF released by eosinophils contributed to the blister formation, which keeps in line with BP severity (51, 58, 71, 72). Giusti et al. also found that the serum concentration of ECP decreased within 0–60 days after treatment, but it did not decrease in patients with subsequent relapse. The cutoff value for the decrease of ECP concentration from baseline to 60 days was 12.8ng/mL, the sensitivity, specificity, positive predictive value, and negative predictive value for judging the clinical remission of BP were 63.2%, 64.3%, 81.1%, and 41.8%, respectively (72).

Eosinophils are known to initiate the coagulation cascade at skin level *via* TF, which in BP promotes the recruitment of eosinophils in skin lesions and the expression of matrix metalloproteinases (MMP)-9, making the coagulation cascade become an auxiliary mechanism that is involved in blister formation. Moreover, serum and BF levels of F1+2 and D-dimer ascend in patients with a more severe disease and significantly descend with the remission of the disease (57, 58, 73). The detection of D-dimer has increased the diagnostic evidence of chronic urticaria in clinics (74). After excluding the influence of other diseases, the indicative effect of D-dimer on refractory BP is worth further exploring.

### Others

Several experimental studies have proved the contribution of the classical pathway of a complement cascade to the formation of BP blisters, C5a produced by complement cascade could recruit and activate mast cells to initiate a downstream inflammatory cascade reaction (75). The deposition of autoantibodies and complement C3 along the dermis-epidermis junction is the diagnostic criteria of BP. Interestingly, a more abnormal complement activation is present in more active patients. The complement-fixation assay is a simple method to detect the complement activation by autoantibodies *in vitro*, which could reflect the BP activity (76). Due to the maturity and simplicity of this clinical assay, a larger sample of clinical trial will be necessary.

On the other hand, the activation of mast cells in the lesions is the earliest event in the formation of BP blister. It cannot only secrete inflammatory cytokines to promote subsequent infiltration of inflammatory cells such as neutrophils in skin lesions, but also release mast cell protease (MCP)-4 to activate major proteases such as MMP-9 that cause BP tissue damage (77). Trypsin in serum and BF, as reliable indicators of mast cell activation, seems to be consistent with the disease activity (78, 79), but more research is required to obtain a high-level evidence.

Modern techniques such as RNA sequencing help identify potential BP biomarkers. MiR-1291 is an endogenous non-coding RNA, which is also a possible biomarker of some autoimmune disorders. A recent study has found that MiR-1291 significantly increase in active patients, and the serum relative expression could reflect the BP activity, the sensitivity and specificity were 75.56% and 81.03%, respectively (80). The implementation of these techniques may not only help to find more potential biomarkers of diseases but also improve our understanding of the pathogenesis of BP. Some clinical characters are also linked to relevant aspects of the BP. At baseline, patients with generalized diseases and neurological diseases such as dementia have a higher risk of relapse (21). It is necessary to assess whether the patients have neurological diseases or a wide range of disease areas as soon as possible to take more positive and reasonable measures.

In summary, both innate immunity and adaptive immunity play important roles in the pathogenesis of BP. Although a large number of laboratory parameters are related to refractory BP, we recommend that especially anti-BP180 NC16A IgG, anti-BP180 NC16A IgE, total IgE, the count of peripheral blood eosinophils, ECP, D-dimer, F1+2, IL-5,CXCL-8, CCL17, and IL-10 are the most promising indicators for severity, based on clinical relevance, repeatability (i.e., more than three studies from different centers showing the same correlation), and feasibility, and their value as biomarkers of disease severity deserves further investigation. Some potential markers such as IL-1β, IL-6, TNF-α, etc. are not specific enough, so they should be interpreted more carefully. However, due to the small number of large-scale studies on biomarkers for treatment resistance or relapse, the level of evidence is still very low, and more research is needed. Having said that, several large-scale studies have provided evidence of the clinical efficacy of biomarkers reflecting the risk of disease relapse or treatment resistance. Detecting BP180NC16AIgG antibody levels at baseline, serum levels of IL17, IL-23, CXCL10, CCL17, BAFF, APRIL, sCD40L, MMP-9, and ECP during treatment, and DIF or BP180NC16A IgG ELISA on the day ready to stop the treatment may help clinicians to identify the patients who are prone to relapse and take more active treatment and management measures to reduce the possibility of relapse., but it should be clear that complex autoantibody and cytokine disorders exist in BP, and it is often not accurate to use a biomarker alone. More large-scale clinical research is needed to explore new biomarkers or verify promising biomarkers that have been found, and the joint detection of these biomarkers is more meaningful to reflect the disease status.

## Emerging Biologics for BP

Currently, no biologics have been approved for BP, and the treatment mainly depends on traditional immunosuppressive therapies. However, high-dose of glucocorticoids and immunosuppressants cause obvious side effects, and are associated with a high relapse rate, precision medicine of refractory BP attracts much attention. With the emergence of biomarkers, more and more biologics targeting the pathogenic mechanism are entering the experimental stage or being used in the treatment of refractory BP in the clinic. We will focus on these biologics.

### Biologics Targeting Pathogenic Antibodies

Pathogenic antibodies including BP180IgG and IgE are used as tools to identify refractory BP, and in some case reports, biologics specifically targeting them have shown great advantages in managing refractory BP.

#### Rituximab

Rituximab is a monoclonal antibody targeting the specific CD20 transmembrane glycoprotein of mature B cells. It causes B cell depletion and antibodies reduction through antibody-dependent cytotoxicity (ADCC), complement-dependent cytotoxicity (CDC), and direct induction of apoptosis. Rituximab was originally used for non-Hodgkin’s lymphoma and has been recommended by the international panel of experts for the first-line treatment of pemphigus (81, 82).

In recent times, Rituximab has been gradually applied to refractory BP. First-line combination therapy with rituximab and corticosteroids could significantly improve the complete remission rate of patients on the basis of reducing the dose of prednisone and not increasing the incidence of adverse reactions. Now, the dose of Rituximab for BP treatment has not been specified, almost all clinical applications use the recommended dose for non-Hodgkin’s lymphoma or rheumatoid arthritis, that is, intravenous infusion of Rituximab 375 mg/m^2^ per week for four weeks or 1,000 mg per week for two consecutive weeks (83).

It is worth noting the relapse and adverse reactions under the treatment of Rituximab. CD20 is not expressed on long-living antibody-producing plasma cells, even the peripheral blood B cells achieve complete consumption and the autoantibodies disappear, patients could only achieve temporary and partial remission, so it is very important to observe the tendency of relapse and to find a suitable maintenance treatment (84). Low peak BAFF levels and the increased proportion of memory B cells before B-cell recovery are helpful to identify BP patients who are prone to relapse or resistant to Rituximab (85). In addition, infusion-related adverse reactions are common and serious adverse events are rare, there are occasional neutropenia, skin toxicity (leukoclastic vasculitis and Stevens-Johnson syndrome, etc.), systemic infection, and even death (86–88). IVIg combined with Rituximab in the treatment of refractory BP could help patients to rebuild the immunomodulatory function to reduce the incidence of adverse events and achieve lasting remission (89). As a steroid protective agent, Rituximab has benefited some patients with refractory BP and brought a higher remission rate, but it is necessary to monitor the hemogram, blood pressure, and temperature, and take care of the infection and infusion-related adverse reactions.

#### Omalizumab

As the first targeted drug approved by the FDA for the treatment of chronic urticaria (74), Omalizumab was successfully cured a patient with refractory BP for the first time in 2009 (90), because it could prevent IgE from interacting with FcϵR I on mast cells and other effector cells to reduce the release of inflammatory mediators by binding to the Fc region of free IgE, downregulating of FcϵR I expression and dissociating the IgE- FcϵR I complex.

A total of 16 studies have reported the efficacy of Omalizumab in the treatment of refractory BP. In these studies, most patients showed elevated total IgE and eosinophil levels and resistance to immunosuppressive therapy, that is, intractable pruritus or extensive skin lesions were difficult to control, or had complications such as osteoporosis and infections. After they were treated with Omalizumab, their itching, new blisters, and eosinophil count were significantly improved at the earliest within a week, the lesions basically disappeared, and the dose of glucocorticoid gradually decreased after an average of 3 months. Most patients could benefit from subcutaneous injection of 300 mg Omalizumab every 4 weeks as a steroid protective agent or monotherapy, with varying maintenance time (up to 18 months), and no serious adverse reactions (36, 38, 90–102). In the absence of large-scale clinical trials, the recommended dose of Omalizumab is based on its use in asthma or chronic urticaria. During the period of treatment, peripheral blood eosinophil counts and FcϵR I have been linked to response to Omalizumab, and followed the disease activity. Patients with a continuous downregulation of Fcϵ RI expression and eosinophil count are more likely to show a satisfactory response to Omalizumab (38, 99).

In the current case reports, the conclusions about Omalizumab and Rituximab for refractory BP are encouraging, but their efficacy and safety have not been tested in clinical trials, which is essential for eliminating bias and determining their options in the treatment of BP. Recently, an open-label, single group design, phase 3 clinical trial evaluating the efficacy and safety of Rituximab combined with Omalizumab in the treatment of BP is underway (NCT04128176).

### Biologics Targeting Complements

Complement cascade reaction is involved in BP blister formation, biologics specifically blocking it may be beneficial to the treatment of BP.

#### BIVV009

BIVV009 (sutimlimab) is a humanized IgG4 monoclonal antibody, and specifically inhibits the first components subcomponent (C1s) of the complement system to interfere with the classical pathway of complement cascade. The safety and activity of BIVV009 for the treatment of BP have been tested in phase 1 clinical trial (NCT02502903) involving 10 active BP subjects. The results showed that 60 mg/kg infusion of BIVV009 four times a week was enough to inhibit the classical complement pathway as well as safe and tolerable in this elderly population. Only mild to moderate adverse events such as headache and fatigue were reported (103).

Given the success of phase I trials, FDA designated BIVV009 as an orphan drug of BP in August 2017, thus promoting future clinical development (75). A prospective, randomized, double-blind, placebo-controlled phase 1 clinical trial (NCT02502903) with a larger sample continues.

#### Avdoralimab

Avdoralimab is a specific antibody against C5aR1, which is safe for the treatment of solid tumors and rheumatoid arthritis. Karsten et al. have previously demonstrated that C5aR1 mediates the anti-BP180 IgG-induced pathogenicity, while C5aR2 has a protective effect (104). Therefore, more specific blocking of the C5a-C5aR1 axis by Avdoralimab is expected to be used in the treatment of BP. Therefore, 40 BP patients are expected to participate in an open-label, multicenter, randomized, parallel-group phase 2 clinical trial (NCT04563923).

### Biologics Targeting Th2 Axis

Dupilumab, a monoclonal antibody against IL-4Rα, not only blocks the signal transduction mediated by IL-4 and IL-13, but also inhibits the secretion of IL-31 by eosinophils (101). It has been approved for the treatment of atopic dermatitis and chronic rhinosinusitis with moderate to severe asthma and nasal polyposis. In most patients with BP, the frequency of cells producing IL-4 and IL-13 in blood and lesions increases, and decreases as the disease improves. Dupilumab is also an effective inhibitor of Th2-related chemokines CCL17, CCL18, CCL22, and CCL26, which fluctuate synchronously with the disease severity. Therefore, Dupilumab could specifically block the pathogenic effects of these cytokines in BP (105). Recently, Dupilumab has been successfully used in refractory BP, especially for the relief of intractable itching (106).

In 2018, Dupilumab monotherapy successfully treated a patient with refractory BP for the first time (105). It also relieves that itching could not be controlled by Omalizumab in the treatment of BP (101). Abdat et al. reported the study with the largest sample. A total of 13 patients with refractory BP initially accepted the approved administration regimen for atopic dermatitis, that is, Dupilumab 600 mg was injected subcutaneously for the first time, and then 300 mg was injected subcutaneously every two weeks, but some patients could only achieve partial control of the disease, so the frequency of medication was mostly changed to once a week. Six of them received Dupilumab monotherapy, seven patients received a combination of glucocorticoid or methotrexate with Dupilumab for an average of 5 months. A total of 84.6% of the patients achieved remission of the disease, and there was no obvious adverse effect. Although 53.8% of the patients were cleared of the disease, 42.9% of them received Dupilumab more frequently than every two weeks. Wherein, the weekly frequency of treatment seems to be more conducive to remission (107).

Currently, a multicenter, randomized, double-blind, parallel-group, placebo-controlled clinical trial (NCT04206553) is in progress to evaluate the efficacy and safety of Dupilumab in adult patients with BP.

### Biologics Targeting Eosinophils

As the main infiltrating cells of BP lesions and the main effector cells of blisters formation, eosinophils are involved in the pathogenesis of BP in many ways, and provide several biomarkers indicating the severity and refractory of BP. Targeting eosinophils is a candidate therapeutic strategy for BP.

#### Bertilimumab

Bertilimumab is a completely human monoclonal antibody targeting eotaxin-1 (CCL-11), while eotaxin-1 is mainly involved in the recruitment of eosinophils from the peripheral circulation to the skin lesion in BP. The use of Bertilimumab is designed to reduce eosinophil infiltration in BP lesions. An open, single-group, phase 2 clinical trial (NCT02226146) has been conducted to study the safety, efficacy, and pharmacodynamics of Bertilimumab in patients with newly diagnosed moderate to generalized bullous pemphigoid. The study included nine subjects with moderate to severe BP who were treated with Bertilimumab as a steroid protector for four weeks. Bertilimumab was well tolerated in all nine subjects. The BPDAI score decreased by 81%, and no serious drug-related adverse events were reported. Based on these results, FDA has granted an orphan drug designation to Bertilimumab in BP.

#### Benralizumab

Benralizumab is a humanized IgG1 κ monoclonal antibody against the IL-5R α subunit, which acts by blocking the downstream signaling of IL-5. It induces target cell killing mediated by NK cells through ADCC, which leads to the decrease of eosinophils and basophils in circulation. Considering the pathogenic role of IL-5 and eosinophils in BP, a multi-country, randomized, double-blind, parallel-group, placebo-controlled phase 3 clinical trial (NCT04612790) has just begun to investigate the effectiveness of Benralizumab for BP.

### Biologics Targeting Th17 Axis

Th17 axis takes part in the blister formation and drug resistance in BP relapse. The patients who are prone to relapse may be the indications for biologics targeting it.

#### Ustekinumab

Ustekinumab is a humanized monoclonal antibody targeting the p40 subunit shared between IL-23 and IL-12. IL-23 often has a synergistic effect with IL-17 in BP relapse (108). To study the efficacy and safety of Ustekinumab combined with super-effective topical corticosteroids in the treatment of BP, an open-label, single-group design phase 2 clinical trial is in progress (NCT04117932).

#### Tildrakizumab

Tildrakizumab is an IL-23 inhibitor that specifically inhibits the p19 subunit of IL-23, thereby reducing its activity. It has been approved for the treatment of adult plaque psoriasis by the FDA (109). Recently, an open-label, single-group design phase 1 clinical trial (NCT04465292) has been conducted to evaluate the efficacy of Tildrakizumab for BP.

Obviously, biologics are promising therapeutic options for patients who are resistant to standard therapy. Rituximab, Omalizumab, and Dupilumab have been successfully used in clinic, and clinical trials of new biologics targeting different mechanisms have been gradually carried out. Furthermore, for the elderly with a high incidence of BP, it is prone to have contraindications of glucocorticoid and immunosuppressive, such as hypertension, arteriosclerosis, diabetes, osteoporosis, and cardiorenal insufficiency, and chronic recurrent course requires long-term and high-dose use of glucocorticoid, which increases the risk of adverse events. Although the local use of glucocorticoids is considered to be relatively safe, it is indeed difficult to implement for extensive lesions. For these patients, appropriate biologics should be available as a first-line therapy regimen by comprehensively evaluating the basic condition, so as to bring better prognosis for patients.

### Markers for Prognosis

The mortality rate of BP patients one year after diagnosis is 9.3%–41% (110). Clinical features and biomarkers of patients that indicate the risk of death have been gradually discovered. Individual factors, such as advanced age, low Karnofsky score (≤40), comorbidity groups of the nervous system (including Parkinson’s syndrome, dementia, and stroke), chronic basic diseases (such as heart failure and chronic kidney disease), and iatrogenic factors including long-term hospitalization and high-dose corticosteroids alone are associated with the increased risk of death. On the contrary, Statins and topical glucocorticoids or low-dose glucocorticoids combined with immunomodulators are protective factors associated with a reduced risk of death (9, 26, 110–116). Moreover, there are a few studies on biomarkers related to the risk of death. High levels of anti-BP180 autoantibodies (≥61 U/mL) at diagnosis have to do with an increased risk of death (26, 110). High erythrocyte sedimentation rate (ESR) and low serum albumin levels are also correlated with the risk of death in BP, but they could not be used as special biomarkers to predict the prognosis of BP, because they are easily influenced by other disorders (115). During treatment, more attention should be paid to the above signals indicating poor prognosis in order to better manage refractory BP.

## Conclusion

Although there is increasing interest in finding reliable indicators for different aspects of BP, the level of evidence supporting existing biomarkers is still very low. We advocate researchers to conduct large-scale multicenter clinical trials as much as possible, and actively explore better detection methods to identify or confirm potential biomarkers. For refractory BP patients indicated by clinical manifestations or biomarkers and elderly patients with severe adverse reactions to high-dose glucocorticoids, biologics becomes the promising treatment, some of them have been of benefit to refractory BP patients. More randomized controlled trials are needed to determine their efficacy, safety, and availability for the treatment of patients with BP. All in all, monitoring and targeting these meaningful factors will play an important role in the management of refractory BP.

## Author Contributions

TZ, BP, and SG conceived this paper. TZ wrote this manuscript. All authors contributed to the article and approved the submitted version.

## Conflict of Interest

The authors declare that the research was conducted in the absence of any commercial or financial relationships that could be construed as a potential conflict of interest.

## Publisher’s Note

All claims expressed in this article are solely those of the authors and do not necessarily represent those of their affiliated organizations, or those of the publisher, the editors and the reviewers. Any product that may be evaluated in this article, or claim that may be made by its manufacturer, is not guaranteed or endorsed by the publisher.
